# Depressive symptoms among survivors of Ebola virus disease in Conakry (Guinea): preliminary results of the PostEboGui cohort

**DOI:** 10.1186/s12888-017-1280-8

**Published:** 2017-04-04

**Authors:** Mamady Mory Keita, Bernard Taverne, Sékou Sy Savané, Laura March, Morifodé Doukoure, Mamadou Saliou Sow, Abdoulaye Touré, Jean François Etard, Moumié Barry, Eric Delaporte, the PostEboGui Study Group, M. Barry, M. Cissé, M. S. Diallo, S. B. Diallo, D. Kassé, N. F. Magassouba, M. S. Sow, I. Savané, L. Koivogui, A. Ayouba, E. Delaporte, A. Desclaux, J. F. Etard, B. Granouillac, S. Izard, A. K. Keita, C. Kpamou, S. Leroy, L. March, P. Msellati, M. Peeters, B. Taverne, A. Touré, S. Baize, L. Abel, C. Delmas, C. Etienne, C. Lacabaratz, C. Lévy-Marchal, Y. Lévy, H. Raoul

**Affiliations:** 1 Psychiatric Unit, Donka National Hospital, University Medical Center of Conakry, BP 925, Conakry, Guinea; 2 Institut de Recherche pour le Développement; IRD-UMI 233/INSERM U 1175, Montpellier University, Montpellier, 911, Avenue Agropolis, BP 64501, 34394 Montpellier CEDEX 5, France; 3 Centre Régional de recherche et de formation à la prise en charge clinique de Fann (CRCF), University Teaching Hospital (CHU) of Fann, Dakar, Senegal; 4 Infectious Diseases Department, Donka National Hospital, University Medical Center of Conakry, BP 925, Conakry, Guinea; 5 Department of Public Health, Conakry University, Conakry, Guinea

## Abstract

**Background:**

The 2013–2016 West African Ebola outbreak infected 28,616 people and caused 11,310 deaths by 11 May 2016, across six countries. The outbreak has also resulted in the largest number of EVD survivors in history—over 17,000. Guinea was declared Ebola-free on 1 June 2016. Reports from the outbreak documented 3814 cases resulting in 2544 deaths and 1270 survivors. EVD survivors face various neuropsychological and psycho-affective alterations that have not been fully identified yet. This study aims to document the depressive symptoms among adult survivors in Guinea.

**Methods:**

Depressive symptoms were investigated using the French version of the Center for Epidemiologic Studies-Depression Scale (CES-D) administered to all adult survivors (≥ 20 years) participating in the PostEboGui study and receiving care in Conakry. The study was combined with a clinical consultation by a psychiatrist at the Donka National Hospital in Conakry that ensured adapted care was provided when needed.

**Results:**

Overall, 256 adult participants receiving care in Conakry participated in this study: 55% were women, median age 31 years [IQR: 26–40]. The median time since the Ebola Treatment Center (ETC) discharge was 8.1 months [IQR: 4.1–11.7]. 15% had a score above the threshold values indicating psychological suffering (15% for men and 14% for women). 33 people (16 women and 17 men) met with the psychiatrist, which resulted in the diagnosis of 3 cases of post-traumatic stress disorder (PTSD), 3 cases of mild depression, 13 cases of moderate depression, and 11 cases of severe depression, including 1 with kinesthetic hallucinations and another with visual hallucinations, and 1 with suicidal ideation and 3 with attempted suicide. Severe depression was diagnosed between 1 and 19 months after ETC discharge. The various identified forms of depression responded favorably to conventional drug therapies and cognitive behavioral therapy.

**Conclusion:**

Long-term follow-up for EVD survivors will be necessary to understand the evolution of these pathologies. In the current post-epidemic context, these cases underscore the need to strengthen mental health diagnostic systems and treatment on a national scale.

**Electronic supplementary material:**

The online version of this article (doi:10.1186/s12888-017-1280-8) contains supplementary material, which is available to authorized users.

## Background

The 2013–2016 West African Ebola outbreak infected 28,616 people and caused 11,310 deaths by 11 May 2016, across six countries (Sierra Leone, Liberia, Guinea, Mali, Nigeria, and Senegal). The outbreak has also resulted in the largest number of EVD survivors in history—over 17,000. Guinea was declared Ebola-free on 1 June 2016. Reports from the outbreak documented 3814 cases resulting in 2544 deaths and 1270 survivors [1].

Ebola virus disease (EVD) is a major traumatic experience, for both the individual and the collective, with many consequences that are still far from being fully known. In the medical field, various somatic manifestations have been reported among survivors, both immediately following the disease [2, 3], and in the long term [4].

Findings of the teams who provided care to patients during this outbreak, backed up by knowledge gained during previous outbreaks [5], quickly led to a consensus on the need to include mental health in the care needed for EVD survivors [6]. Recommendations for follow-up and medical and social care for EVD survivors have been identified, including for mental health [7]. However, little is still known about the consequences of EVD on survivors’ mental health [8].

The aim of this study is to describe the mental health of adult EVD survivors in Guinea by studying depressive symptoms in order to promote care for EVD survivors and to help assess mental health needs in the post-Ebola context in Guinea.

## Method

The PostEboGui cohort is a multi-centric prospective study that aims to: (i) provide access to care for EVD survivors (all medical consultations, complementary exams, and drugs are provided for free), and (ii) to study the clinical, immuno-virological, psychological, and socio-anthropological effects of the disease for a 24-month period following discharge from the Ebola Treatment Center (ETC). Participants were enrolled in four sites (Conakry, Macenta, N’Zérékoré, and Forécariah) between 23 March 2015 and 11 July 2016. These included all persons who were at least one year old, live in Guinea, had EVD confirmed by laboratory exams and were declared clear of the virus in the blood when leaving the ETC, and who have given their consent [9].

In this article, data are from: (1) standardized questionnaires administered during a PostEboGui study visit, and (2) patients’ clinical observations collected during psychiatric consultations.

### Data collection

#### Standardized questionnaires

Patients’ clinical and virological characteristics were collected at their inclusion then during their follow-up in the study. A socio-demographic questionnaire was also administered at the time the participant enrolled in the study. This questionnaire can identify the number of people in the participant’s household unit or family who were suspected of being exposed to EVD or who died from it.

Psychological status was investigated using the French version of the Center for Epidemiologic Studies Depression Scale (CES-D) [10, 11] to all persons aged 20 years and older. The evaluation scale was administered to all participants during their first consultation as part of the PostEboGui study.

The CES-D scale assesses the subject’s mood by asking how often he or she experienced physical or psychological symptoms or exhibited behaviors associated with anxiety or depression in the previous week [12]. The CES-D scale is a self-administered questionnaire of 20 items; the respondent assesses each item using a Likert-type scale with 4 possible answers, ranging from 0 to 3. The total score is calculated by adding the response values, totaling between 0 and 60. High scores correspond to more severe depression; the threshold selected as an indicator of depression was 17 for men and 23 for women [11]. Due to the high illiteracy rate, for this study, the questionnaire was either filled out by the physician during the first medical consultation or by a socio-anthropologist investigator at the end of the first consultation in the PostEboGui program; the questions were translated by the physician or investigator into the language used by the patient. Given the health emergency conditions in which this study was set up, it was not possible to conduct a cross-cultural validation of the CES-D scale. The tool’s translation, back-translation, reproducibility, structural validity, and sensitivity to change were not assessed in the context of this study. However, the instrument’s internal consistency is consistent with the literature (Cronbach’s alpha: 0.86).

Results of the CES-D score were reported in terms of the time elapsed since discharge from the ETC with the goal of testing the hypothesis of greater psychological suffering immediately following the disease. The CES-D score results were also reported in terms of two aspects that have been hypothesized as possibly influencing the onset of psychological suffering: (i) the subjective perception of health status and the current situation, given that the perception of poor health or impoverished living conditions could result in a higher probability of psychological suffering, and (ii) EVD infection of other members of the household unit, since multiple experiences of illness or death in the family could result in a higher probability of psychological suffering.

#### Patients’ clinical observations

People with a score higher than the critical threshold were referred to a clinical consultation conducted by a psychiatrist to establish the diagnosis and to propose psychotherapeutic care. Treatment could also be provided to patients whom the physician deemed required a psychiatric consultation, independently of the CES-D result. Diagnoses were identified and categorized based on the ICD-10, Version: 2008 (http://apps.who.int/classifications/icd10/browse/2008/en#/).

### Study population

The study population is comprised of all persons aged 20 years and older participating in the PostEboGui study, who were receiving care at the Conakry site and who completed the CES-D questionnaire.

### Statistical analyses

For this analysis, the CES-D score was divided into two categories; depending on whether the score was below or above the threshold (adjusted for sex), the participants were divided into two groups: those whose score was below or equal to the threshold (no characteristic symptoms of depression) and those with a score higher than the threshold (presence of characteristic symptoms of depression). Comparisons were performed using a Fisher exact test for categorical variables and a non-parametric Mann-Whitney test for continuous variables. Data were analyzed using Stata 13/SE software (StataCorp LP, Texas, US).

### Ethical considerations

The protocol was approved by the Research Committee of the National Ebola Response Coordination (28 January 2015) and the National Ethics and Health Research Committee in Guinea (015/CNERS/15, 2015/02/16) and by the French Ethics Committees Inserm/CEEI (no. 15–201; 16 February 2015) and IRD/CCDE (2015). All participants provided written informed consent.

## Results

### Descriptive epidemiology

**Fig. 1:**
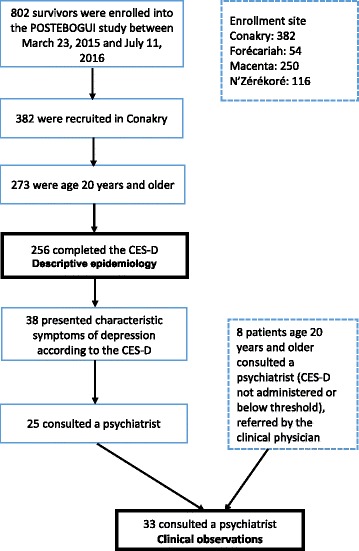
Flow chart describing the study population

On 11 July 2016, the cohort had recruited 802 participants, including 382 in Conakry, 273 of whom were age 20 years or older. The results presented here involve 256 adults included in Conakry from 23 March 2015 to 23 February 2016 who completed the CES-D (cf. Fig. 1).

The main characteristics of the study population are presented in Table 1. Of the 256 people in this population, 138 are women (54%) and 118 are men (46%); the median age is 32 years [IQR: 26–40]. The median time elapsed since the ETC discharge is 8.1 months [IQR: 4.1–11.7]; 19.1% of the people were discharged from the ETC less than 3 months before joining the study (and their psychological evaluation); 19.5% 1 year or more before joining.

**Table 1:** Comparison of clinical and socio-demographic characteristics of PostEboGui participants, age 20 and older, enrolled in Conakry, based on the presence or lack of characteristic symptoms of depression as defined by the CES-D. *N* = 256

	No characteristic symptoms of depression	Characteristic symptoms of depression	Total	*P*-Value^c^
	*n* = 218	*n* = 386	*N* = 256	
	85%	15%		
	n(%)	n(%)	n(%)	
	median [IQR]	median [IQR]	median [IQR]	
Sex				
Male	100 (84.7%)	18 (15.3%)	118	0.862
Female	118 (85.5%)	20 (14.5%)	138	
Age				
	31.36 [25.7;40.4]	32.06 [25.5;36.8]	31.56 [25.7;39.9]	0.908
Time between ETC discharge and psychological evaluation, in months				
	7.92 [4.3;12.0]	8.17 [3.0;10.8]	8.10 [4.1;11.7]	0.374
Time between ETC discharge and psychological evaluation				
Less than 3 months	39 (79.6%)	10 (20.4%)	49	0.088
3–6 months	42 (93.3%)	3 (6.7%)	45	
6–9 months	46 (85.2%)	8 (14.8%)	54	
9–12 months	45 (77.6%)	13 (22.4%)	58	
One year or more	46 (92.0%)	4 (8.0%)	50	
Situation dominated by the disease^a^				
No	185 (86.0%)	30 (14.0%)	215	0.607
Yes	29 (82.9%)	6 (17.1%)	35	
Situation dominated by poverty^a^				
No	71 (83.5%)	14 (16.5%)	85	0.569
Yes	143 (86.7%)	22 (13.3%)	165	
Situation dominated by the effects of stigmatization^a^				
No	201 (86.6%)	31 (13.4%)	232	0.152
Yes	13 (72.2%)	5 (27.8%)	18	
Situation dominated by a return to normal life^a^				
No	52 (83.9%)	10 (16.1%)	62	0.678
Yes	162 (86.2%)	26 (13.8%)	188	
Number of Ebola cases in the household and family circle^b^				
Interviewee only	47 (83.9%)	9 (16.1%)	56	0.096
2–5 Ebola cases	86 (91.5%)	8 (8.5%)	94	
6+ Ebola cases	82 (81.2%)	19 (18.8%)	101	
Number of Ebola cases in the household and family circle^b^				
	4.00 [2.0;8.0]	6.00 [1.5;8.5]	4.00 [2.0;8.0]	0.496
Number of deaths in the household and family circle^b^				
No deaths	57 (81.4%)	13 (18.6%)	70	0.177
1–4 deaths	106 (89.8%)	12 (10.2%)	118	
5+ deaths	52 (82.5%)	11 (17.5%)	63	
Number of deaths in the household and family circle^b^				
	2.00 [0.0;4.0]	2.00 [0.0;5.0]	2.00 [0.0;5.0]	0.990

*Abbreviations*: *IQR* Interquartile Range, *ETC* Ebola Treatment Center

^a^6 missing data

^b^4 missing data

^c^Comparisons were performed using a Fisher exact test for categorical variables and a non-parametric Mann-Whitney test for continuous variables

Among the 256 people who completed the CES-D scale questionnaire, 38 people (15%) had a score higher than the threshold values. We found that 15.3% of men presented with depressive symptomatology as defined by the CES-D compared to 14.5% of women; however, this difference is not statistically significant (p. 0.862).

The percentage of people with a CES-D score above the critical threshold ranged between 20% for people who were discharged from the ETC 3 months earlier and 8% for people who were discharged more than 12 months earlier; although these rates vary depending on how much time had elapsed since ETC discharge, these differences are not significant.

The people who perceive themselves as “still sick” or for whom their current situation is “dominated by stigmatization” have a CES-D score above the threshold more frequently; however, the observed differences are not statistically significant.

The hypothesis that there is a greater percentage of people with signs of psychological suffering among people who had several sick or deceased family members seems to be refuted. The percentage of people with a CES-D score above the threshold is higher among those who were the only ones who had EVD in their household unit and family (16% had CES-D scores above the threshold when they alone were ill versus 15% of patients who had sick family members; 19% had scores above the threshold when there were no deaths versus 15%); the observed differences are not statistically significant.

### Clinical observations

Thus, 33 people (age ≥ 20 years, 16 women and 17 men) had a clinical consultation with a psychiatrist; the median age was 31 years on the day of the consultation. Among these adults, 25 presented with a depression score above the threshold value and 8 were referred for a psychiatric consultation by the clinical physician, based on clinical evidence, even though the depression score was below the threshold.

The main characteristics of people seen in a consultation and the psychiatric diagnoses are presented in Table 2 and Table 3. Among the severe depression cases, 1 was accompanied by kinesthetic hallucinations and another by visual hallucinations; 1 person presented suicidal ideation, and 3 people attempted suicide. Post-traumatic stress disorder (PTSD) was diagnosed in 3 people, isolated in 2 people, and combined with severe depression in another. Severe depression was diagnosed between 1 and 19 months after ETC discharge. The 3 people who attempted suicide were discharged from the ETC 5, 11, and 12 months earlier.

**Table 2:** Main characteristics of people seen in a psychiatric consultation

	Women	Men	Total
N	16	17	33
Age (years) (median)	32	31	31
Time since discharge from the Ebola Treatment Center			
Less than 6 months	4	4	8
6–9 months	2	5	7
9–12 months	2	5	7
12 months or more	8	3	11
Diagnosis			
Mild depression	–	3	3
Masked depression	–	1	1
Moderate depression	9	4	13
Severe depression	6	5	11
PTSD (Post-Traumatic Stress Disorder)	1	2	3
No clinical signs	–	3	3

**Table 3:** Distribution of diagnoses according to the number of months since ETC discharge

Time since ETC discharge	< 6 months	6–9 months	9–12 months	> 12 months	Total
Diagnosis					
Mild dep.	–	2	1	–	3
Masked dep.	–	1	–	–	1
Moderate dep.	3	3	1	6	13
Severe dep.	3^a^	–	5^a^	3^b^	11
PTSD	#	–	1	1	3
No signs	1	1	–	1	3

^a^with attempted suicide

^b^combined with PTSD

The symptoms described by these people include the usual signs of the depressive syndrome: sleep disorders (problems falling asleep, awaking at night); anorexia-type eating disorders; sad expression; diminished vitality and feelings of worthlessness; psychomotor slowing; exacerbation of addictive behaviors (alcohol, cannabis); thoughts of death; and sometimes attempted suicide.

In addition, various complaints associated with depressive symptoms were reported by 18 of the 33 people. These were primarily muscle and joint pain (11), headaches (7), eye pain or decreased visual acuity (5), ear pain or decreased auditory acuity (4), and memory impairment (3). These complaints, despite sometimes being described as extremely incapacitating, do not appear to be more common in people with symptoms of severe depression.

Lastly, the percentage of people whose score was positive and who did not come for a consultation is 34% (13/38). When comparing the characteristics of patients who did not come for a consultation with those who did come, it was noted that those who did not come: were more likely to be female (77% versus 40%), did not speak French (55% versus 9%), resided far from Conakry (42% versus 30%), had a low level of education (55% had never been to school versus 33%), and were frequently unemployed (92% versus 71%). However, because of the low numbers in our sample, we were unable to determine if these differences are significant. The remote locations of survivors’ residence and the language barrier appear to be reasonable hypotheses for understanding why many consultations were not conducted. These elements seem to reflect a process of social selection in using the psychiatric consultation, even if the PostEboGui project covered any costs related to this consultation.

## Discussion

### Limitations of the study

The quantitative aspects of this study are based on the use of the CES-D scale. This tool was selected following discussions with psychiatrists from the Donka National Hospital and French psychiatrists with experience in health crises in Africa and based on the study team’s previous experience in assessing depression among people living with HIV in Africa (Senegal and Côte d’Ivoire). Use of the CES-D score has been validated in various social and cultural contexts [13–15].

Various biases may have influenced data collection using the questionnaire. The CES-D is designed to be used as a type of self-assessment. The population’s high illiteracy rate and the difficulty of translating the questionnaire into the country’s four main local languages made it impossible to use it in that way. Therefore, the questionnaire was completed by a physician or a socio-anthropologist investigator who received advanced training in order to standardize their understanding of the questionnaire and how to administer it. The questionnaire includes no medical terms and only uses standard vocabulary that is familiar, thus limiting the risks of incorrect translations. The CES-D scale was used as a tool for screening and not for diagnosis. In February 2016, WHO recommended the use of the Patient Health Questionnaire for depression (PHQ-9). This questionnaire closely resembles the CES-D. The few research teams that have worked in post-Ebola mental health in Sierra Leone and Liberia used various tools (for example, the Kessler Psychological Distress Scale) that all present comparable limitations in terms of validity and adaptation to cultural and social contexts.

The scores obtained relate to the person’s psychological condition during the week before filling out the questionnaire, without specifically taking into account any social support that could help the person or a recent event not directly related to the EVD episode. Lastly, the CES-D score is an assessment tool for depression; it does not cover all symptoms or conditions that EVD survivors may present. Nevertheless, the depression assessment score could provide an initial overall assessment of mental health. It enables care providers to prioritize who is most at risk of depression so they may be referred for a psychiatric consultation in a context where mental health care delivery is weak.

Comparing results obtained from the CES-D scale with the clinical consultations identified a few cases of false positives and false negatives. Analysis of these differences does not fall within the scope of this study since its objective did not include validating the specificity or sensitivity of this tool. These observations point to the value in combining an epidemiological approach with a clinical approach.

Our study did not identify any statistically significant association between depressive signs and various factors that are hypothesized as being linked to depression (seriousness of the disease, number of deaths in the family, sequelae, etc.). Rather, these various factors are related to cases of depression among patients who had a psychiatric consultation. This finding is possibly related to a lack of sensitivity in the tools or a low statistical power related to the size of the survey population.

Another limitation of the study is the lack of a control group or information on the prevalence of depression in the general population. Also, it is impossible to attribute causality to the findings or to know whether they are more frequent in EVD survivors than in the general population. Case-control studies would be adequate to check the real burden of Ebola virus as a trigger or risk factor associated with depression in their population.

### Prevalence of psychological suffering

All the EVD survivors attested to the extent of their psychological suffering during their stay in the ETC but also in the early stages following their ETC discharge. Knowledge gained from previous outbreaks indicates the extent of psychological harm [16, 17]. However, comparing the prevalence reported during previous outbreaks or in the few available studies on the current outbreak is difficult due to the diversity of observations and measuring tools used.

During the 1995 Kikwit outbreak in the Democratic Republic of the Congo, De Roo et al. [5] reported that 61% of the 34 participants in their study presented “psychological consequences” from the disease, but the exact nature of the consequences is not described. Nor is the time period between recovery and their onset.

A recent study of 74 survivors conducted in Sierra Leone in 2015 in the Moyamba District reported that “48% [of them] present signs and symptoms of distress, several weeks and months after discharge, putting them at risk of experiencing different levels of mental health disorders” [18]; this study does not provide precise information on the time elapsed since discharge from the ETC. Another study, also in Sierra Leone, conducted among 81 people in the Kenema District found depression among 35%, up to 4 months after the ETC discharge [19].

In Liberia, the preliminary results of the ongoing study conducted by the Prevail III project on neurological sequelae report a 49% prevalence of depression, for a sample of 82 people discharged from a treatment center at least 6 months before [20]. Another study conducted by an MSF team in Monrovia describes a 40% prevalence of depression for a sample of 136 people discharged from an ECT more than 3 months before and 12% for major or severe depression associated with PTSD [21].

A study was conducted by Qureshi et al. [2] in the Conakry region involving 105 EVD survivors between March and August 2014. These people were divided into two groups depending on the time elapsed since their recovery, 0–90 days and 91–201 days, with the average time since recovery at 103 days ±47 days; 32% of those in the first group and 38% in the second group complained of mood disorders and 1% of “self-perceived depression.”

Overall, the percentage of people included in the PostEboGui study in Conakry presenting depressive symptoms appears significantly lower than in the observations reported recently in Sierra Leone and Liberia. This gap is possibly related to the use of different evaluation tools; it may also be related to different socio-cultural contexts and the social impact of the outbreak. The much higher number of people affected in Sierra Leone and Liberia than in Guinea probably had different repercussions depending on the regions and communities.

### Severity of psychological suffering

The clinical consultation shows that nearly all the participants with a CES-D score above the critical threshold presented various depressive symptoms, including some very serious ones. In the context of the PostEboGui cohort, those identified as “a suicidal risk” were directed to psychiatric services where they could receive adapted psychotherapeutic care. The care package that was set up proved to be in line with WHO recommendations, which were drawn up afterwards [7].

Some cases of severe depression with attempted suicide following EVD were reported in earlier studies [22]. Recently in Liberia, Bowen et al. [20] identified 2 “actively suicidal” people among the 82 people receiving follow-up. Serious forms of depression can be diagnosed several months after discharge from the ETC (diagnosis 21 months after the ETC discharge for one participant of the PostEboGui cohort), the causal link with the EVD episode is therefore more difficult to confirm.

### Efficacy of medical-psychological care and the health care system’s capacity to meet needs

Depressive symptoms identified among EVD survivors did not appear to present a particular specificity in terms of semiology nor in how they responded to treatment. The therapeutic care suggested by the psychiatrists as part of follow-up in the PostEboGui study was not specific, but it nevertheless required the use of drugs in the most serious cases that usually must be prescribed solely by specialist physicians.

Most of survivors were not able to receive effective psychosocial support due to the lack of psychiatrists, clinical psychologists, and social workers in the country. Having a sufficient number of qualified health professionals present to provide medical and psychological care and the availability of suitable drugs is currently a major limitation in Guinea: at the end of 2015, there were only 5 psychiatrists (including 1 child psychiatrist), 13 generalists trained in psychiatry, and 10 psychologists from NGOs [23]. Generally, and independently of the EVD epidemic, delivery of mental health care and treatment is very low in Guinea and is almost exclusively limited to the capital city Conakry. Moreover, health professionals (nurses and physicians) are not accustomed to referring people who present signs of psychological suffering to psychologists or psychiatrists. In most cases, only individuals presenting major psychotic conditions, in urban areas, are referred to specialized services.

## Conclusion

Survivors of EVD face various forms of somatic and psychological sequelae; psychological alterations have not been fully identified and described yet. Our exploratory study of 256 EVD survivors in Guinea, for a median time period of 8 months, showed that 15% of them presented depressive symptoms; the clinical consultation showed that these sometimes major symptoms have significant repercussions on these people’s capacity for social reintegration. Long-term follow-up for EVD survivors will be necessary to understand the evolution of these pathologies. In the current post-epidemic context, these observations underscore the need to develop and strengthen mental health diagnostic systems and care on a national scale.

In addition to EVD survivors, special attention should focus on people who were not sick themselves but who were affected by the disease when one or more relatives became sick or died. Epidemiological studies in mental health aimed at assessing the epidemic’s psychological impact on families affected by EVD would provide a better understanding of the disease’s overall impact and help gear social and health interventions to promote collective resilience to the epidemic.

## Additional files

Additional file 1: Center for Epidemiologic Studies Depression Scale (CES-D). (PDF 59 kb)

Additional file 2: Questionnaire CES-D Adultes >20 ans. (PDF 159 kb)
