# Supplementary material for: Depressive symptoms among survivors of Ebola virus disease in Conakry (Guinea): preliminary results of the PostEboGui cohort
Source: BMC Psychiatry. 2017 Apr 4;17:127. doi: 10.1186/s12888-017-1280-8 (PMC5381039; doi:10.1186/s12888-017-1280-8)
Supplement: Supplementary file 1 — Center for Epidemiologic Studies Depression Scale (CES-D). (PDF 59 kb) [file 12888_2017_1280_MOESM1_ESM.pdf]

Patient Name:_______________________________ Date:__________________

Center for Epidemiologic Studies Depression Scale (CES-D)

Instructions: Below is a list of the ways you might have felt or behaved. Please tell me how often you have felt this way **during the past week**.

	Rarely or none of the time (less than 1 day)	Some or a little of the time (1-2 days)	Occasionally or a moderate amount of time (3-4 days)	Most or all of the time (5-7 days)
1. I was bothered by things that usually don't bother me.				
2. I did not feel like eating; my appetite was poor.				
3. I felt that I could not shake off the blues even with help from my family or friends.				
4. I felt I was just as good as other people.				
5. I had trouble keeping my mind on what I was doing.				
6. I felt depressed.				
7. I felt that everything I did was an effort.				
8. I felt hopeful about the future.				
9. I thought my life had been a failure.				
10. I felt fearful.				
11. My sleep was restless.				
12. I was happy.				
13. I talked less than usual.				
14. I felt lonely.				
15. People were unfriendly.				
16. I enjoyed life.				
17. I had crying spells.				
18. I felt sad.				
19. I felt that people disliked me.				
20. I could not get "going."				

Scoring the CES-D

In scoring the CES-D, a value of 0, 1, 2 or 3 is assigned to a response depending upon whether the item is worded positively or negatively.

For items 1-3, 5-7, 9-11, 13-15, 17-20 the scoring is:

- Rarely or none of the time (less than one day) = 0
- Some or a little of the time (1-2 days) = 1
- Occasionally or a moderate amount of time (3-4 days) = 2
- Most or all of the time (5-7 days) = 3

Items 4, 8, 12, 16 are reverse scored as follows:

- Most or all of the time (5-7 days) = 0
- Occasionally or a moderate amount of time (3-4 days) = 1
- Some or a little of the time (1-2 days) = 2
- Rarely or none of the time (less than 1 day) = 3

Possible range of scores is 0 to 60, with the higher scores indicating the presence of more symptomatology.

Citation: Radloff LS: The CES-D Scale: a self-report depression scale for research in the general population. Applied Psychological Measurement 1:385-401, 1977.
