# Supplementary material for: Depressive symptoms among survivors of Ebola virus disease in Conakry (Guinea): preliminary results of the PostEboGui cohort
Source: BMC Psychiatry. 2017 Apr 4;17:127. doi: 10.1186/s12888-017-1280-8 (PMC5381039; doi:10.1186/s12888-017-1280-8)
Supplement: Supplementary file 2 — Questionnaire CES-D Adultes >20 ans. (PDF 159 kb) [file 12888_2017_1280_MOESM2_ESM.pdf]

# Questionnaire CES-D Adultes > 20 ans

**Identifiant Patient :** |__| |__|__|__| |__|__|__|__|

N° centre N° patient Code anonyme

Visite n° : |__| |__|__| Date de la visite : |__|__| |__|__| |__|__|__|__|

Date du questionnaire : |__|__| |__|__| |__|__|__|__|

| Durant la semaine écoulée | Jamais très rarement | Occasion-nellement | Assez souvent | Fréquemment tout le temps |
|---|---|---|---|---|
| | moins de 1 jour | 1 à 2 jours | 3 à 4 jours | 5 à 7 jours |
| 1. Vous avez été contrarié(e) par des choses qui d'habitude ne vous dérangent pas | 0 | 1 | 2 | 3 |
| 2. Vous n'avez pas eu envie de manger, vous avez manqué d'appétit | 0 | 1 | 2 | 3 |
| 3. Vous avez eu l'impression que vous ne pouviez pas sortir de la tristesse, même avec l'aide de votre famille et de vos amis | 0 | 1 | 2 | 3 |
| 4. Vous avez eu le sentiment d'être aussi bien que les autres | 3 | 2 | 1 | 0 |
| 5. Vous avez eu du mal à vous concentrer sur ce que vous faisiez | 0 | 1 | 2 | 3 |
| 6. Vous vous êtes senti(e) déprimé(e) | 0 | 1 | 2 | 3 |
| 7. Vous avez eu l'impression que toute action vous demandait un effort | 0 | 1 | 2 | 3 |
| 8. Vous avez été confiant(e) en l'avenir | 3 | 2 | 1 | 0 |
| 9. Vous avez pensé que votre vie était un échec | 0 | 1 | 2 | 3 |
| 10. Vous vous êtes senti(e) craintif(ve) | 0 | 1 | 2 | 3 |
| 11. Votre sommeil n'a pas été bon | 0 | 1 | 2 | 3 |
| 12. Vous avez été heureux(se) | 3 | 2 | 1 | 0 |
| 13. Vous avez parlé moins que d'habitude | 0 | 1 | 2 | 3 |
| 14. Vous vous êtes senti(e) seul(e) | 0 | 1 | 2 | 3 |
| 15. Les autres ont été hostiles envers vous | 0 | 1 | 2 | 3 |
| 16. Vous avez profité de la vie | 3 | 2 | 1 | 0 |
| 17. Vous avez eu des crises de larmes | 0 | 1 | 2 | 3 |
| 18. Vous vous êtes senti(e) triste | 0 | 1 | 2 | 3 |
| 19. Vous avez eu l'impression que les gens ne vous aimaient pas | 0 | 1 | 2 | 3 |
| 20. Vous avez manqué d'entrain | 0 | 1 | 2 | 3 |
| Total des points | | | | |
| Score final | | | | |

Si le score du total des questions 1 à 20 est > 17 pour les hommes et > 23 pour les femmes, parlez-en au médecin référent de la personne.
